# Large-scale waterproof and stretchable textile-integrated laser- printed graphene energy storages

**DOI:** 10.1038/s41598-019-48320-z

**Published:** 2019-08-14

**Authors:** Litty V. Thekkekara, Min Gu

**Affiliations:** 1 Laboratory of Artificial-Intelligence Nanophotonics, School of Science, RMIT University, Melbourne, 3001 Australia; 2 Centre for Artificial Intelligence Nanophotonics, School of Optical-Electrical and Computer Engineering, University of Shangai for Science and Technology, Shanghai, 200093 China

**Keywords:** Electronic properties and devices, Electronic properties and devices, Supercapacitors, Supercapacitors

## Abstract

Textile integrable large-scale on-chip energy storages and solar energy storages take a significant role in the realization of next-generation primary wearable devices for sensing, wireless communication, and health tracking. In general, these energy storages require major features like mechanical robustness, environmental friendliness, high-temperature tolerance, inexplosive nature, and long-term storage duration. Here we report on large-scale laser-printed graphene supercapacitors of dimension 100 cm^2^ fabricated in 3 minutes on textiles with excellent water stability, an areal capacitance, 49 mF cm^−2^, energy density, 6.73 mWh/cm^−2^, power density, 2.5 mW/cm^−2^, and stretchability up to 200%. Further, a demonstration is given for the textile integrated solar energy storage with stable performance for up to 20 days to reach half of the maximum output potential. These cost-effective self-reliant on-chip charging units can become an integral part for the future electronic and optoelectronic textiles.

## Introduction

*E-textiles or smart garments*^1,2^ are a branch of wearable technologies which attained much attention due to its vast potentialities like the incorporation of electronic devices, energy storages and antennas with textiles. The technology enables the development of self-reliant next-generation wearable devices applicable to wireless communications^3,4^, health sensing and monitoring^5,6^, and light-emitting devices^7,8^ which can find applications in smart cities, remote areas, telecommunications, and biomedical industries.

The realization of these self-powered technologies requires the support of energy harvesters like a solar cell or piezogenerator^9,10^. But the intermittent nature of these energy harvesters demands the association of energy storages which are cost-effective, durable, high-performance, have mechanical strength for flexibility and stretchability, non-toxic and inexplosive^11^. Currently, cumbersome coin-cells or pouch cell lithium-ion batteries are in use for this purpose, which is either glued or stitched into the garments^12^. Another common energy storages used for the e-textiles are based on fibers or yarns^13–17^ extended towards largescale fabrication capabilities^18,19^ incorporate electrodes, separators, and electrolyte into a single fiber. However, the low surface area of this category can result in low energy storage capabilities^11^ which leads to the requirement of heavy bundles of the fibers to be assembled to achieve required energy storage to support the e-textiles.

Printed energy storages^20,21^ can be utilized as an alternative to the other integrable energy storages for the powering of wearable technologies due to the lightweight, and compactness. The primary methods used for energy storage electrode fabrication involve screen printing^22,23^ and inkjet printing^24^. But these methods add to the additional steps and time in the fabrication, low mechanical robustness and contributes to the extra cost, which can have a negative impact on the large-scale production of energy storage integrated e-textiles.

On the other hand, laser printing^25^ using the optimized irradiation conditions is a single step optical lithographic fabrication method useful for nano to large-scale strcutures^26,27^. The use of low-dimensional materials like activated carbons and graphene as the electrode materials for these energy storages along with the suitable electrolytes can solve the energy storage-related issues of the e-textiles^14,28^. Recent reports on the development of stretchable laser-printed graphene supercapacitors^29^ up to 150% is a promising development for the wearable technologies, which in turn relies on the self-healing properties of graphene oxides (GOs)^30^.

Even though there are several reports of the printed energy storage prototypes^10,31^, the knowledge of the largescale fabrication capabilities of functional energy storages and their adaptability with wearables are still limited. Here we demonstrate the fabrication and characterization of largescale waterproof and stretchable textile integrated laser-printed graphene energy storages in a dimension of 100 cm^2^ in 3 minutes of fabrication time which belongs to the category of supercapacitors and extend the study of these supercapacitors for the textile integrated solar energy storage (Supplementary Notes).

## Results and Discussion

### Large-scale laser-printed graphene supercapacitors

**Figure 1:**
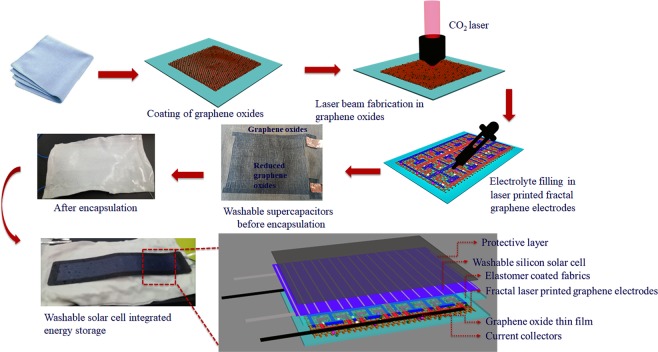
Schematic of the fabrication steps for the largescale waterproof laser-printed graphene solar energy storages.

The schematic of the entire process to form the waterproof laser-printed graphene energy storage, which extends towards the formation of graphene solar energy storage was given in Fig. 1. In the initial stage, an elastomer solution made from polydimethylsiloxane (PDMS) was coated on one side of the fabrics to attain a waterproof nature for the Nylon fabrics and left until the coating was dried (Methods). The other side of these fabrics were paint coated with the graphene oxide (GO)/Matte binder solution to form thin films of thickness 3 µm. The presence of the binder medium provided a waterproof nature for the GOs.

Further, trials on the reduction of the coated GO/Matte thin film on fabrics with a consideration for avoiding the damages for the underlying textiles were done using different types of laser beams varying from the continuous wave (CW) to femtosecond (fs) pulses for obtaining a uniform electrical conductivity and porosity conditions for the energy storage electrodes since these two factors were essential in the formation of the energy storage capacitance^31^. A detailed study on the generation of porosity in laser-printed graphenes using a CW carbon dioxide (CO_2_) laser beam which induces a photothermal reduction and fs laser beam pulses which induces a combination of photothermal and photochemical reduction can be found in our earlier literatures^29,32^.

**Figure 2:**
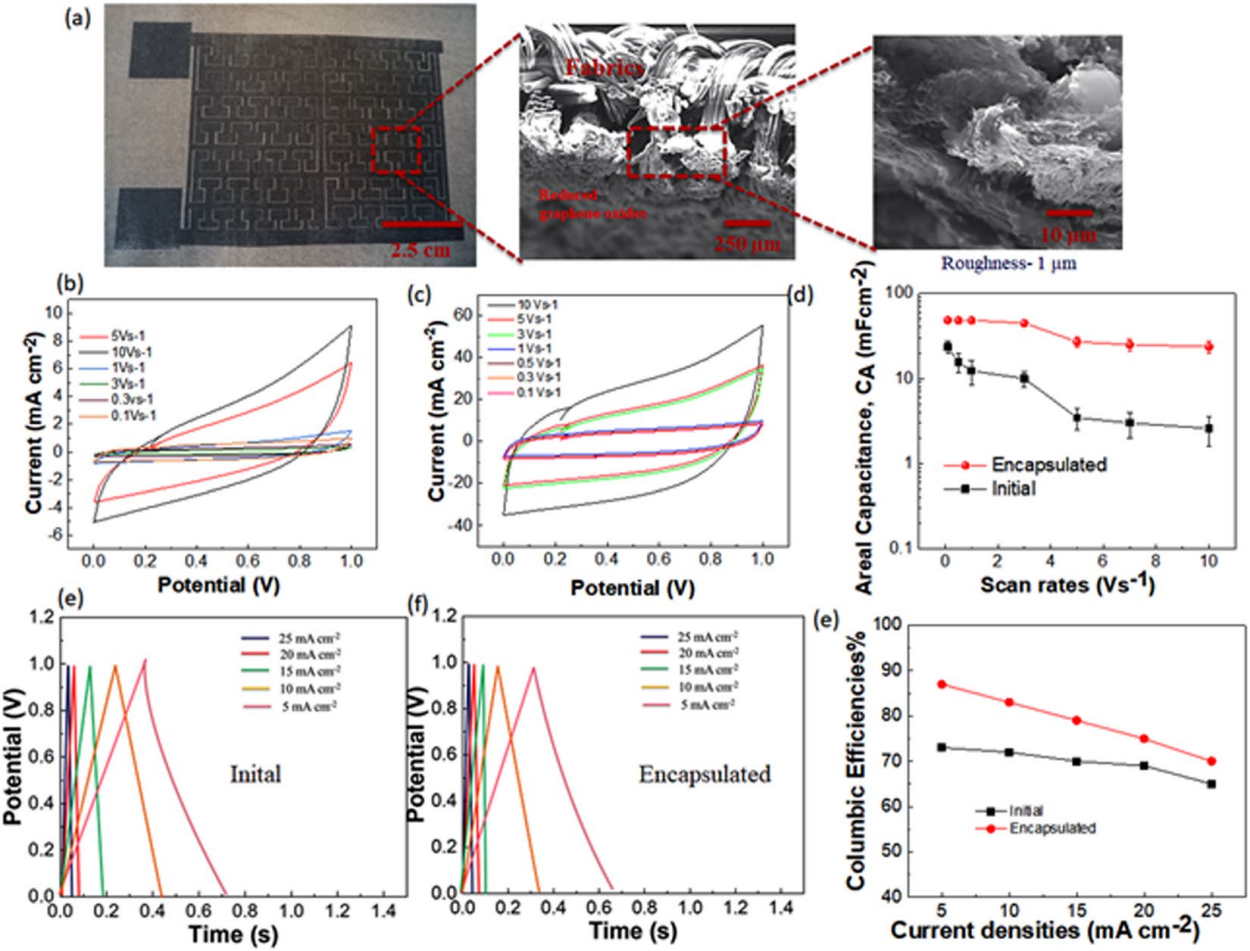
Laser-printed graphene supercapacitors. (**a**) Photo image of the laser-printed graphene fractal electrodes in an area of 100 cm^2^ obtained at a laser beam power of 6 W (Scale-2.5 cm). Highlighted: Scanning electron microscopy (SEM) image of fabrics coated with laser-printed graphene thin films with thickness 10 µm, and the exposed laser-printed graphene due to the photothermal reduction of GO thin films resulting from the removal of water content. (**b**) CV measurements with different scan rates from 0.1 to 10 Vs^−1^ without the encapsulation conditions. (**c**) CV measurements with varying rates of the scan from 0.1 to 10 Vs^−1^ with the encapsulation conditions. (**d**) Areal capacitance of the energy storages calculated from the CV measurements. (**e**) CC measurements with different current densities from 5 to 25 mA cm^−2^ without the encapsulation conditions. (**f**) CC measurements with different current densities from 5 to 25 mA cm^−2^ with the encapsulation conditions. (**g**) Columbic efficiencies for the energy storages calculated from the CC measurements.

Besides, it was observed that the photothermal reduction using a CO_2_ laser beam with an objective of the lower numerical aperture (NA) of 0.35 was suitable for attaining the optimized conditions in comparison to other laser beam interactions with GO/Matte thin film on the fabrics (Fig. 2a). In this study, the optimized Hilbert fractal designs^32^ from our previous studies were used for the formation of supercapacitor electrodes in an area of 100 cm^2^ with an interelectrode distance of 80 µm.

The scanning electron microscopy (SEM) image of the energy storage electrodes of thickness 10 µm from the resultant laser-printed interconnected graphene thin film bound to the fabrics could be seen in Fig. 2a (highlighted). The consequent thickness enhancement in graphene thin films was due to the removal of water molecules along with other functional groups, but from the XPS measurements, it can be seen the peaks for C=O bond are present which are contributed from the binder medium (Supplementary Fig. 1) during the laser beam irradiation using the optimum conditions. Raman measurements (Supplementary Fig. 2a), were used to confirm the optimum photoreduction in the GOs from the presence of G and 2D peaks at 1580 cm^−1^ and 2690 cm^−1^ respectively.

Four-probe measurements were conducted to understand the electrical conductivity of the resultant graphene thin films using irradiation with various laser beam powers (Supplementary Fig. 2b). It can be seen that the threshold condition for the photoreduction of GO/Matte thin films were around 4.5 W. The breakdown in the resultant thin films due to the defect formation in the atomic structure of GOs occurs approximately at 8 W. An optimum electrical conductivity of 20 S/m was obtained for the thin films using the laser beam power of 6 W and was used for the fabrication of the supercapacitor electrodes.

Further, a systematic study on the electrical conductivity of the resultant thin films were performed with several washes using water and drying cycles (average of 50 cycles) of the thin film coated fabrics. It was observed that the electrical conductivity of the thin film after these studies was consistent with the initial conditions. The roughness of the thin film was estimated to be around 1 µm from the atomic force microscopy (AFM) (Supplementary Fig. 3).

Polyvinylalcohol (PVA)/sulphuric acid (H_2_SO_4_) gel electrolyte with an electrochemical window of 1 V was used in the supercapacitor since it can perform at the room conditions, and the system can be dried. Copper tapes were used as the current collectors for these energy storages. Besides, an encapsulation for the supercapacitor was made using another elastomer coated nylon fabrics for the integrability with the e-textiles to meet the environmental conditions. In the encapsulated graphene energy storages, copper wires were used as the current collectors.

### Performance

The electrochemical characterizations were conducted on the obtained supercapacitors with an average of 10 repetitive cycles before and after the encapsulation using cyclic voltammetry (CV), galvanostatic charge-discharge (CC) and electrochemical impedance spectroscopy (EIS) as given in Fig. 2. The measurement details could be found in the *Methods* section. It was seen from the CV measurements that the rectangular behavior of the voltammograms was improved with the encapsulation and indicated the better stability for the electrolyte ionic flow in the supercapacitor under those conditions (Fig. 2b,c). The areal capacitance of the obtained supercapacitors at higher scan rates in both mentioned scenarios was around 49 and 24 mF cm^−2^, respectively (Fig. 2d).

CC measurements were performed on the supercapacitor without encapsulation using current densities from 5 to 25 mA cm^−2^ (Fig. 2e) shows a triangular behavior even at high current densities with a columbic efficiency of 73% at lower current densities. Figure 2f shows the CC measurements on encapsulated supercapacitors, and columbic efficiency was calculated to be around 87% for lower current densities. Coulmbic efficiencies for various current densities under encapsulated and nonencapsulated conditions for the energy storages could be found in Fig. 2g.

Further, EIS measurements were conducted from a frequency range of 10 Hz to 10 kHz to confirm the electrolyte ionic flow in the electrode pores of the supercapacitor. From the Nyquist plot (Supplementary Fig. 4), it was seen that the supercapacitor with an encapsulation had an internal resistance of 1.0 Ω. From these results, it can be seen that the obtained areal capacitance are higher in comparison to the other printed textile-based supercapacitors using screen-printing technology reported in literature^33^.

A shortcoming of these supercapacitors was the lower performance per cm^2^ in comparison to the electrodes made from other laser-printed graphene supercapacitors^32^ due to the addition of nonconductive binding medium even though the total surface area is increased. On the contrary, these energy storage prototype opens a path for further development of the on-chip wearable technology support due to the other favorable features like stretchability and water stability.

### Stretchability tests

**Figure 3:**
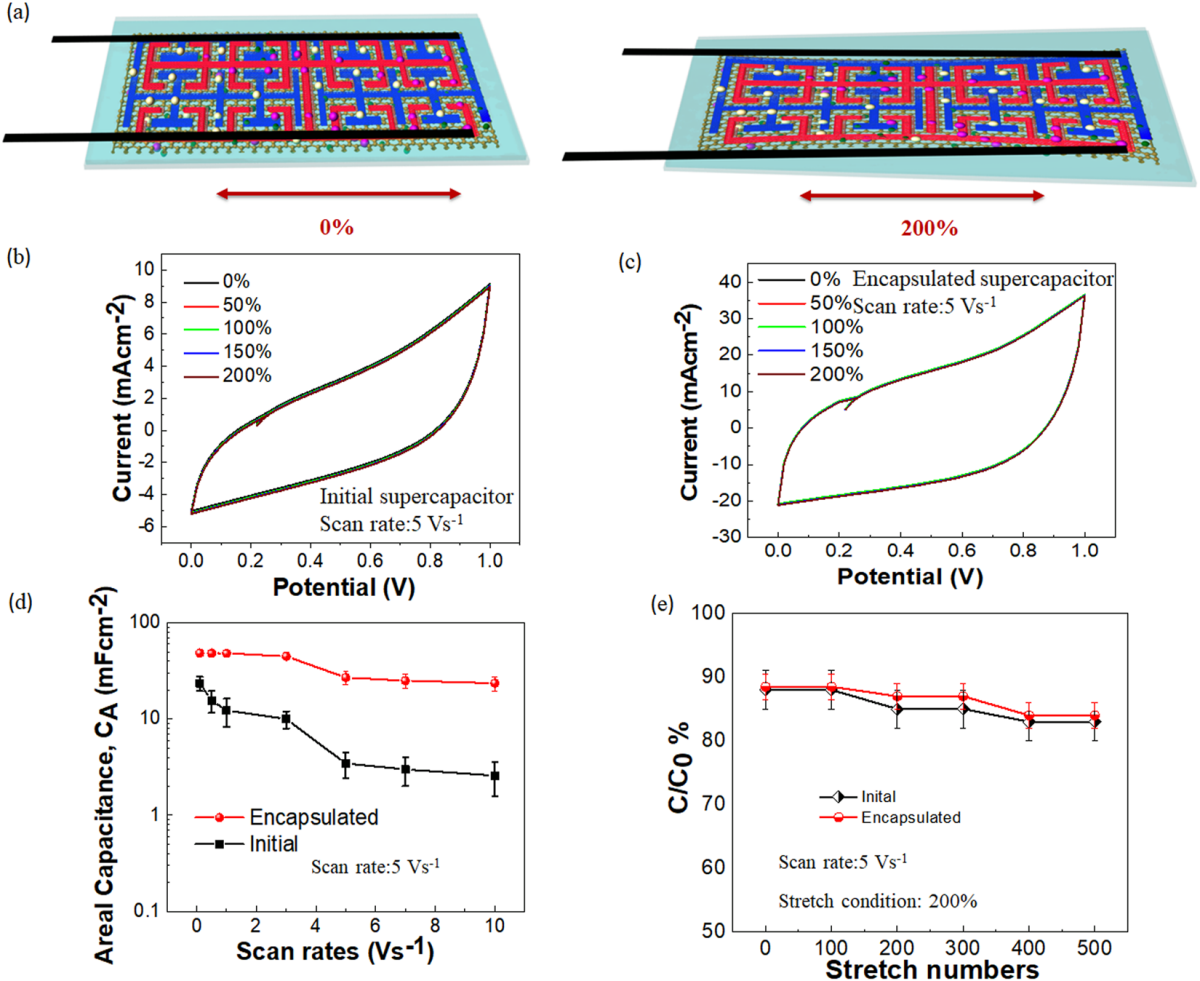
Stretchability tests of the laser-printed graphene supercapacitors. (**a**) Schematic of stretching of laser-printed graphene supercapacitors integrated on textiles. (**b**) CV measurements on laser-printed graphene supercapacitors without encapsulation under different stretchable conditions for a scan rate of 5 Vs^−1^. (**c**) CV measurements on laser-printed graphene supercapacitors with encapsulation under different stretchable conditions for a scan rate of 5 Vs^−1^. (**d**) Areal capacitance of laser-printed graphene supercapacitors with and without encapsulation under different stretchable conditions for a scan rate of 5 Vs^−1^. (**e**) Capacitance retention under the maximum stretchable condition of 200% along the uniaxial direction for a scan rate of 5 Vs^−1^.

An essential requirement for the e-textile energy storages was the mechanical flexiblity^34^. Tests were performed using CV measurements on the fabric integrated supercapacitors for stretchability in the room conditions (Supplementary Fig. 5) and a schematic of the process was given in Fig. 3a. During the CV measurements, a rectangular behavior for the voltagramms under a scan rate of 5 Vs^−1^ for a maximum of 200% stretchability along the uniaxial direction was observed for the fabricated supercapacitors without encapsulation (Fig. 3b) where the maximum stretchability for an encapsulated supercapacitor was slightly less up to 175% due to the presence of additional fabrics. The CV performance of encapsulated energy storage could be found as Fig. 3c.

The areal capacitance during the stretchable conditions was around 3.45 mF cm^−2^ for unencapsulated energy storages and 27 mF cm^−2^ for encapsulated energy storage with a decrease up to 10% at more substantial stretchability (Fig. 3d). The capacitance retention under the maximum stretchable condition of 200% along the uniaxial direction for a scan rate of 5 Vs^−1^ was around 88% for supercapacitors under both situations (Fig. 3e). A comparison for the areal capacitance of various printed stretchable supercapacitors was given in Supplementary Table 1. The performance of the resultant stretchable devices remains the same due to the self-healing properties of reduced graphene oxides^30^.

### Washability and stability

**Figure 4:**
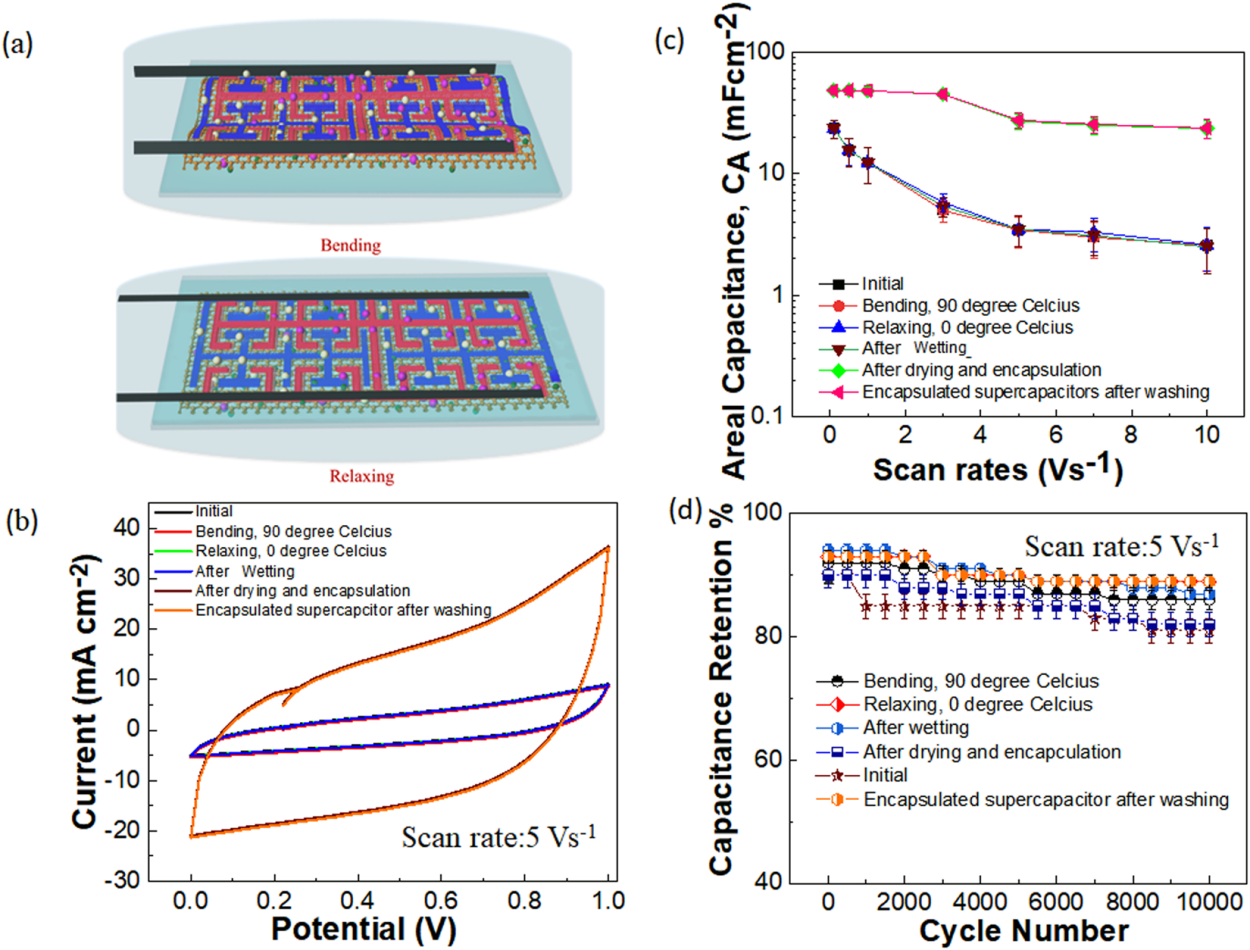
Washability tests for laser-printed graphene supercapacitor. (**a**) Schematic of washing tests of laser-printed graphene supercapacitors integrated on textiles. (**e**) CV measurements on the laser-printed supercapacitor under various conditions of temperature, compression, relaxing, and encapsulations with a scan rate of 5 Vs^−1^. (**c**) Areal capacitance of the laser-printed graphene supercapacitors for different scan rates and under different conditions of temperature, compression, relaxing and encapsulations. (**e**) Capacitance retention of the laser-printed supercapacitor under various conditions of temperature, compression, relaxing, and encapsulations with a scan rate of 5 Vs^−1^.

Cyclic compression tests on the laser-printed graphene supercapacitors under different situations of encapsulation (Supplementary Fig. 6) were performed with the entire supercapacitors submerging in hot water of 90 °C, as shown in Fig. 4a. It was seen that the CV curve for the supercapacitor during the compression condition was lower in comparison to the other states (Fig. 4b) and this was contributed from the induced strain as well as high temperature during the bending situation. Further, it was seen that the areal capacitance for the supercapacitor in the presence of water was higher in comparison with the dried electrolyte containing supercapacitor (Fig. 4c). This behavior was explained from the higher aqueous ionic flow among the electrode pores in the presence of water. It was seen in Fig. 4d that these supercapacitors under the conditions of compression in hot water, relaxing in cold water and encapsulations maintain capacitance retention between 83 to 94% at a scan rate of 5 Vs^−1^ up to 10,000 cycles. In addition, we have performed the normal washing tests in a commercial washing machine up to 50 cycles, and details of the test are given in Methods, and the observed results show capacitance retention of 80%, as shown in Supplementary Fig. 7. These results provided exceptional stability for the large scale laser-printed graphene supercapacitor under simultaneous conditions of mechanical challenges, temperature, and washability.

## Discussion

The simultaneous maintenance of high efficiency during the mechanical tests and water stability enabled the washability of textile integrated on-chip laser-printed graphene energy storages. In addition, the stable performance of these energy storages combined with a solar cell module introduced real-time storage of renewable energies for the e-textiles (Supplementary Notes). Further, these large-scale laminated energy storages open the possibilities for the faster roll-to-roll fabrication to meet the industrial competence with the utilization of advanced laser printing based on multifocal fabrication^35^ and machine learning^36^ techniques. These energy storages will support the next-generation textile based eco-friendly durable and non-exploding self-powered technologies.

## Methods

### Materials

The GOs of concentration 4 mg/ml was purchased from GRAPHNEA. The washable amorphous silicon solar module of dimension 25 × 4 cm^2^ with features of 0.7 W and 1.5 V were purchased from BUHESHUI. Spandex Nylon Lycra Matte fabrics were used as textiles in the studies. One side of textiles was coated with Polydimethylsiloxane (PDMS) elastomer to make it waterproof. Otherside of the textiles was coated with GO/binder medium, which forms the background for the energy storage preparation. Liquitex Matte liquid was used as the binder medium with GOs. The electrolyte used in the studies was polyvinyl alcohol (PVA)/sulphuric acid (H_2_SO_4_).

### Device fabrication

The CO_2_ laser beam was found to be more efficient for the supercapacitor electrode fabrication of fabrics. An area of 100 cm^2^ fractal laser-printed electrodes^32^ in the thin film (prepared from GOs/binder/water mixture) was fabricated using 6 W laser beam power with 80 µm interelectrode distance. 0.3 ml PVA/H_2_SO_4_ electrolyte was dropped on the fabricated electrodes and dried under 60 °C for half an hour to remove the excess water content from the obtained supercapacitors. Copper tapes (3 M) were used as the current collector in the unencapsulated form, and insulated copper wires were used as the current collectors in the encapsulated energy storages. Further, the obtained supercapacitor was encapsulated on the top side of the fabrics and stitched together so that the supercapacitors will not expose to room conditions, and washable amorphous silicon thin film solar module was glued (Gloo fabric glue) to the encapsulated supercapacitor, and aluminum tapes were used as the current collectors (Supplementary Notes).

### Device encapsulation

To avoid the influence of external parameters like moisture in the performance of supercapacitors, we used an encapsulation. The encapsulation was made from PDMS coating on one side of the textile without affecting the stretchability. The final supercapacitor was fabricated on the other side of the PDMS coated textiles, and another piece of PDMS coated textiles were stitched together to form a complete enclosed device. The thickness of PDMS is controlled for improving the comfortability of the textile for wearing.

### Washability tests

The textile integrated supercapacitor was put into a commercial laundering machine (Whirlpool). Each laundering cycle lasted for 30 min, and the agitator started to rotate at a spinning speed of 120 rpm for 10 min. After running each cycle and drying naturally, the electrical outputs after washing at different times were measured.

### Characterizations

Raman spectra of different layers of graphene oxides were studied using a Renishaw Raman spectrometer equipped with an Nd-YAG laser beam of wavelength 514 nm. Scans were performed between 1,000 and 2,700 wavenumbers with a laser spot size of 1 µm. The background-corrected spectrum was normalized by dividing the data by the maximum intensity. SEM images were conducted using Philips XL30 SEM with an operating voltage of 15 keV. Four-probe conductivity measurements on laser-printed graphene films reduced under different irradiation conditions were performed using the Keithley 2400 series source meter wired through four micro-positioners using tungsten probes of tip diameter 5 µm. Measurements were taken by varying the applied voltage between 0.1 V to 4.5 V. The electrical conductivity studies were performed by keeping the probes on each side of current collector copper tapes. The XPS measurements were conducted using the Thermo K-alpha X-ray Photoelectron Spectrometer (XPS) using monochromatic, Micro-focused Al K-α source.

### Electrochemical characterizations

The electrochemical measurements with an average of ten repetitions include cyclic voltammetry (CV), galvanostatic charge-discharge (CC) and impedance measurements (EIS). CV measurements were performed with a step size of 0.1 V from 0 to 1 V; galvanostatic CC measurements were conducted with varying current densities from 5 to 25 mA cm^−2^ and EIS measurements were performed in a frequency range of 10 kHz-10 Hz were conducted using potentiostat station (Gamry 1010) at room temperature. The device volume includes two laser-printed graphene current collector electrodes; laser-printed graphene fractal electrodes and the separator. Stretchability tests were performed using a custom-built setup for CV measurements. Washability tests were conducted in the lab under room and high-temperature conditions using CV measurements. The calculations used in this study was given in Supplementary Notes.

## Supplementary information

Large-scale waterproof and stretchable textile-integrated laser- printed graphene energy storages
