# Supplementary material for: Large-scale waterproof and stretchable textile-integrated laser- printed graphene energy storages
Source: Sci Rep. 2019 Aug 14;9:11822. doi: 10.1038/s41598-019-48320-z (PMC6694168; doi:10.1038/s41598-019-48320-z)
Supplement: Supplementary file 1 — Large-scale waterproof and stretchable textile-integrated laser- printed graphene energy storages [file 41598_2019_48320_MOESM1_ESM.docx]

Large- scale waterproof and stretchable textile-integrated laser- printed graphene energy storages

Litty V Thekkekara^1^ and Min Gu^1,2†^

^1^Laboratory of Artificial-Intelligence Nanophotonics, School of Science, RMIT University, Melbourne-3001, Australia

^2^Centre for Artificial Intelligence Nanophotonics, School of Optical-Electrical and Computer Engineering, University of Shangai for Science and Technology, Shanghai, 200093, China

^†^Corresponding author email id:gumin@usst.edu.cn

**Supplementary notes**

**Materials.** The *PDM,* which was used as elastomers for the fabrics were prepared in the ratio 10:1 ([Sylgard 184](http://scholar.google.com.au/scholar?q=pdms+sylgard+184&hl=en&as_sdt=0&as_vis=1&oi=scholart)). The *Matte medium* which was the binder medium with graphene oxides (GOs) is an aqueous emulsion of polymethyl-methacrylate and polyethylene-glycol (PMMA + PEG). The solution for the coating was prepared in the following ratio: 24 g of GOs, 10 ml of water and 5 ml of binder medium. The chemical solution was stirred for half an hour to obtain a uniform mixture. The mixture was painted on the fabrics and left to dry overnight to form thin films of thickness 3 µm.

The electrolyte used in the studies were made from 1g polyvinyl alcohol (PVA) and 10g sulphuric acid (H_2_SO_4_) which has an electrochemical window 1 V. The mixture was stirred and heated until the solution turned clear and kept in the room temperature.

**Comparison between various laser beam irradiation for the supercapacitor fabrication.** A continuous-wave (CW) laser beam and femtosecond (fs) laser beam pulse were irradiated in the thin film coated fabrics to optimize the supercapacitor electrode fabrication conditions. The CW laser beam consists of carbon dioxide (CO_2_) of wavelength, 10.6 µm, an objective of the numerical aperture (NA) 0.35, Versa)) and varying beam powers from 2.4 to 9 W. The fs laser beam of wavelength 800 nm, the pulse repetition rate of 5 kHz and width of 120 fs with objectives of NA 1.4, 0.95 and 0.75 were used.

From these studies, it can be confirmed that the pulse energy density provided from fs laser beam^1^ of lower pulse repetition rates like 5 kHz in GOs is not sufficient enough for the complete removal of the oxygen groups which can, in turn, affect the energy storage capabilities of the resultant supercapacitors. An optimized irradiation condition using CW was used in the electrode fabrication of the supercapacitor in an area of 100 cm^2^.

**Electrical conductivity calculation.** The bulk conductivity of the obtained thin films was evaluated using the following equation:

*σ*=*l*/*RA* (1)

where *σ* is the electrical conductivity (S/m), *R* is the resistance, *l* is the length of the thin film, and *A* is the area of the thin film.

**Electrochemical calculations.** The specific areal capacitance was calculated from cyclic voltammetry (CV) curves using the formula^2^:

$$C_{A}=\frac{1}{2*S*v*(V_{f}-V_{i})}\int_{V_{i}}^{V_{f}} I\left( V \right)dV (2)$$

where *S* refers to the total surface area (cm^2^) of the active electrodes, $v$ is the volatge sweep rate (in Vs^-1^), *V_f_* and *V_i_* are the potential limits of CV curves and *V* is the voltammetric current (in amperes). $\int_{V_{i}}^{V_{f}} I\left( V \right)dV$ is the integrated area from the CV curves.

Energy density, *E* was calculated using the following formula,

$$E=C_{A}*\frac{\left( \Delta E \right)^{2}}{2*3600} (3)$$

where *ΔE* is the electrochemical window of the electrolyte.

Power density, *P* was calculated using the following formula,

$P=\frac{{(\Delta E)}^{2}}{4*R_{ESR}*A} (4)$

where  *R_ESR_* is the internal resistance of the supercapacitor, and *A* is the area of the supercapacitor.

**Solar energy storages**

Further, the studies were extended towards the on-chip solar energy storages with the addition of a washable thin film amorphous silicon solar cell with the encapsulated laser-printed graphene supercapacitor energy storages using the gluing method (Supplementary Figure 7) for the textile-based wearable technologies. The solar cell performance was analyzed before and after the integration with encapsulated laser-printed graphene supercapacitors. The studies were extended to different time intervals of the solar charging process. The solar charging measurements were studied by a solar simulator (Oriel 3A) under a one-Sun condition (1000 W/m^2^) at the room temperature. The aluminum and copper tapes were connected between solar cell and energy storage, so that charge generated in the solar cell will be simultaneously charged the energy storage. A saturation state was observed depending on the capacity of energy storage to store the charge as well as the ability of the solar cell to generate the charge under the longer light exposure times. The self-discharge was studied in the atmospheric conditions.

The performance of a solar cell module was tested under different periods before and after the energy storage integration as well as washing. It was observed that the solar cell module performance remains constant without much variation from the initial current-voltage output conditions where the obtained short-circuit current (I_sc_) is around 0.35 A and an open-circuit voltage (V_oc_) is about 8.5 V (Supplementary Figure 7a).

From the cyclic voltammetry (CV) measurements on solar energy storages at different scan rates from 1 to 10 Vs^-1^ shows an areal capacitance of 49 mF cm^-2^ before and after washing with cold water (Supplementary Figure 7b) at lower scan rates. The solar charging under one solar condition (1000 W/m^2^) and galvanostatic discharging of supercapacitor up to 800 ms showed a stable performance (Supplementary Figure 7c). The tests were not further continued since the continues exposures on amorphous silicon material in the solar cell module could result in an efficiency degradation. These measurements on solar energy storages after washing with water maintained a similar solar charging and discharging cycles in the given period which further confirms the stability of the fabricated solar energy storages for the use of these self-powered textiles in the routine purposes. Besides, the studies on these storages in the room condition show that it took up to 20 days to reach half of the maximum output potential (V_1/2_) (Supplementary Figure 7d).





**Supplementary Figure 1.** XPS measurements on the laser-printed graphene electrodes.


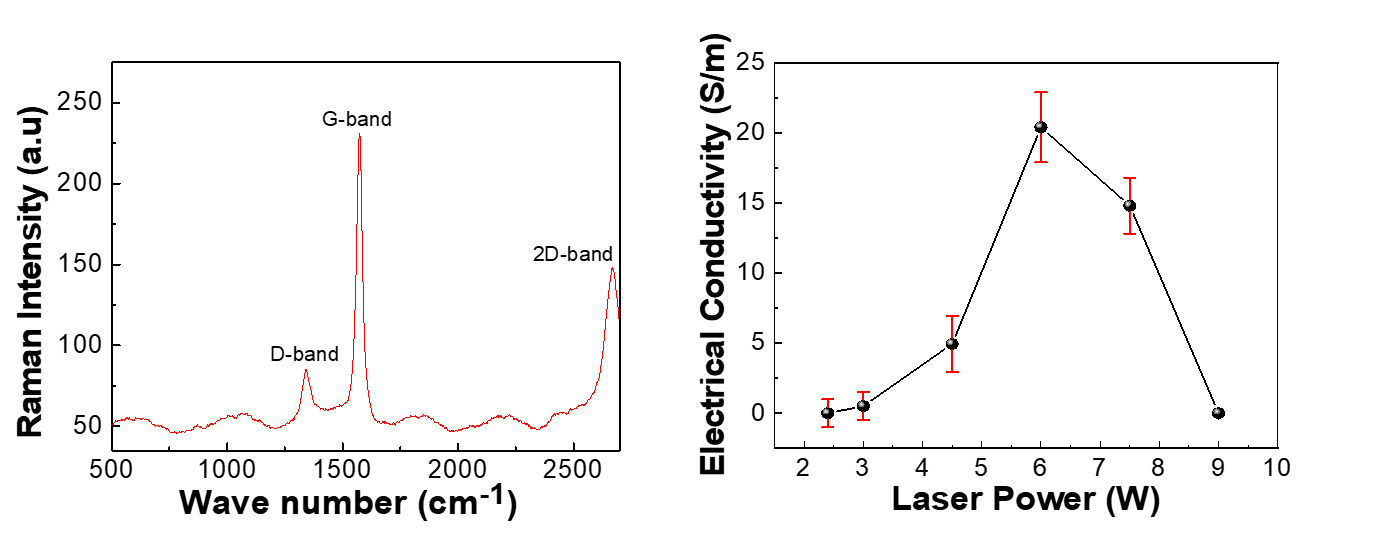


**Supplementary Figure 2. Characterizations of laser-printed graphene film.** (a) Raman spectroscopic image of the laser-printed graphene electrodes. (b) Electrical conductivity measurements on the laser-printed graphene electrodes.


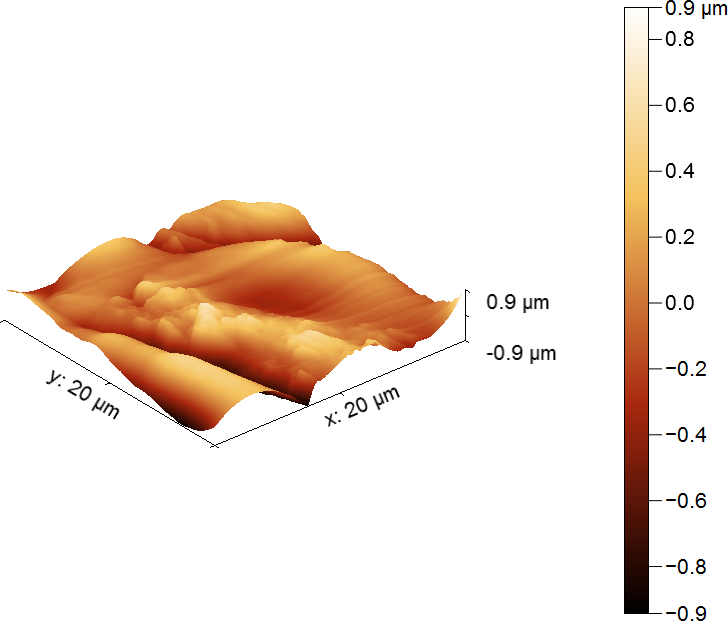


**Supplementary Figure 3**. Atomic force microscopy (AFM) image of the laser-printed graphene thin films shows a roughness of 1 µm.


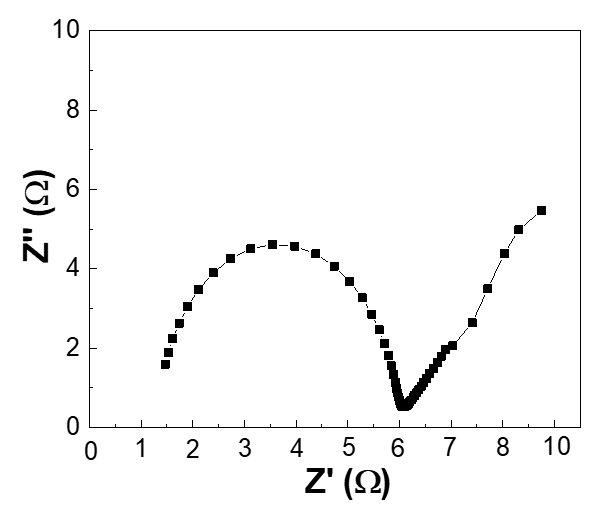


**Supplementary Figure 4.** Nyquist plot of the laser-printed graphene supercapacitor with encapsulation for different frequency scans from 10 Hz to 10 kHz. The ESR of the obtained supercapacitor was confirmed to be 1.0 Ω.


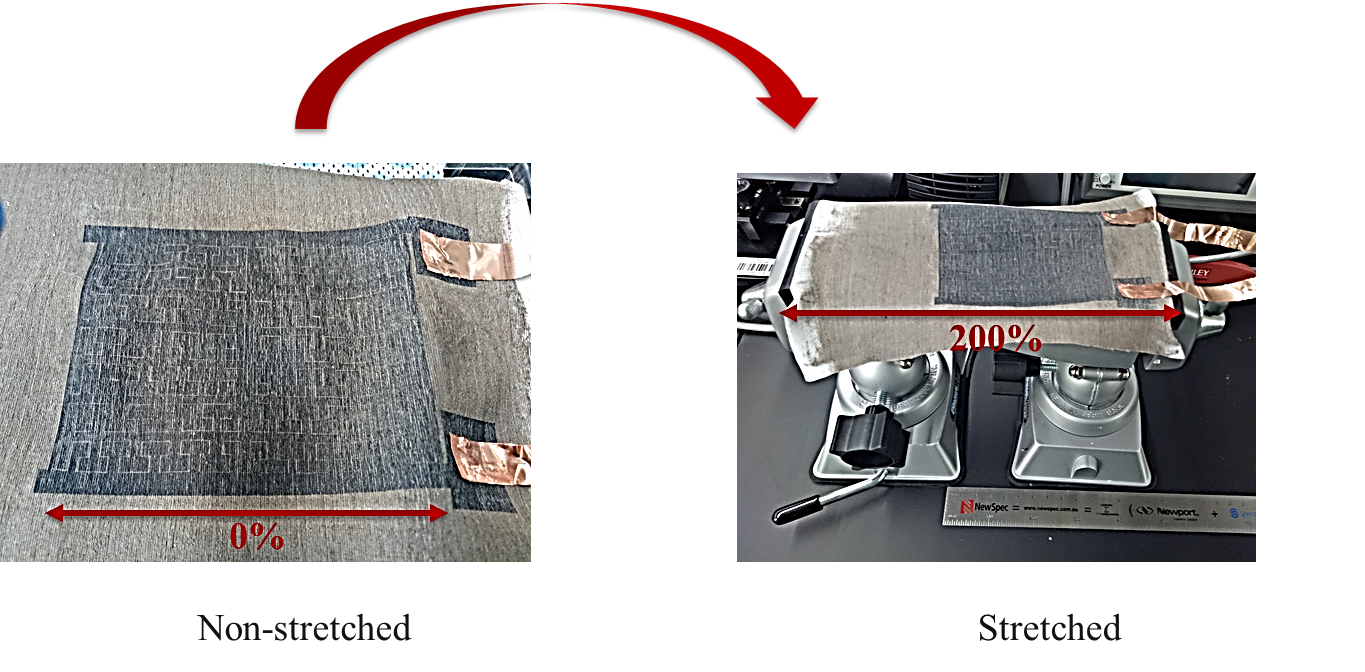

**Supplementary Figure 5.** Image of stretchability measurements on the laser-printed graphene supercapacitor.


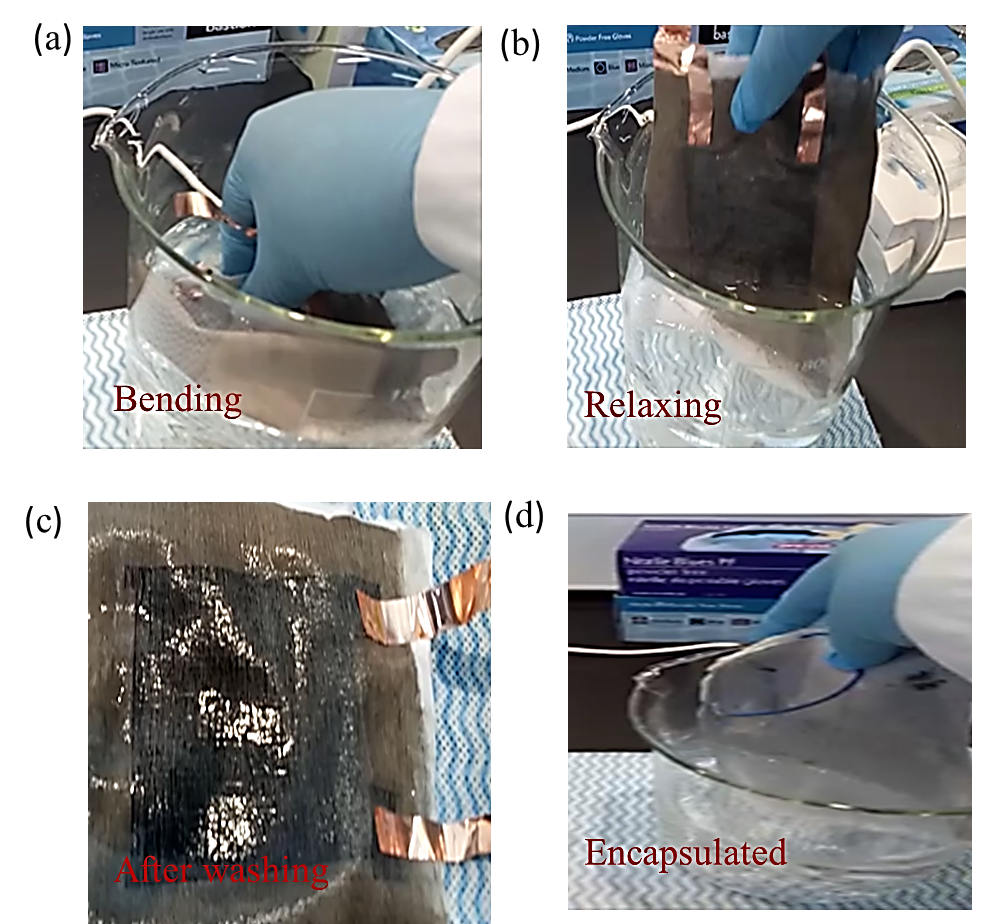


**Supplementary Figure 6.** (a-d) Image of the cyclic compression tests in water for the laser-printed graphene supercapacitor.

**Supplementary Figure 7.** Capacitance retention of the laser-printed graphene supercapacitor during the washing cycles with a scan rate of 5 Vs^-1^.

**Supplementary Table 1.** Performance comparison between laser fabricated stretchable supercapacitors.

| Type | Substrate | Stretchability (%) | Areal  capacitance  (mFcm^-2^) |
| --- | --- | --- | --- |
| Transferred  stretchable LSG  supercapacitor^3^ | PDMS | 50 | 0.65 |
| Two-photon induced graphene supercapacitor^1^ | PDMS | 150 | 0.138 |

Laser reduced- PDMS 60 4

graphene-oxide/ PANI

/Au heterostructures^4^

Laser cut Polyamide 282.5 3.65

Kirgami supercapacitors^5^

Screen printed Cotton 95.6 2.5

supercapacitors^6^

# Screen printed PET - 1.3

# supercapacitors^7^

# Screen printed Silk - 19.2

# supercapacitors^8^

#

This work Nylon textiles 200 49


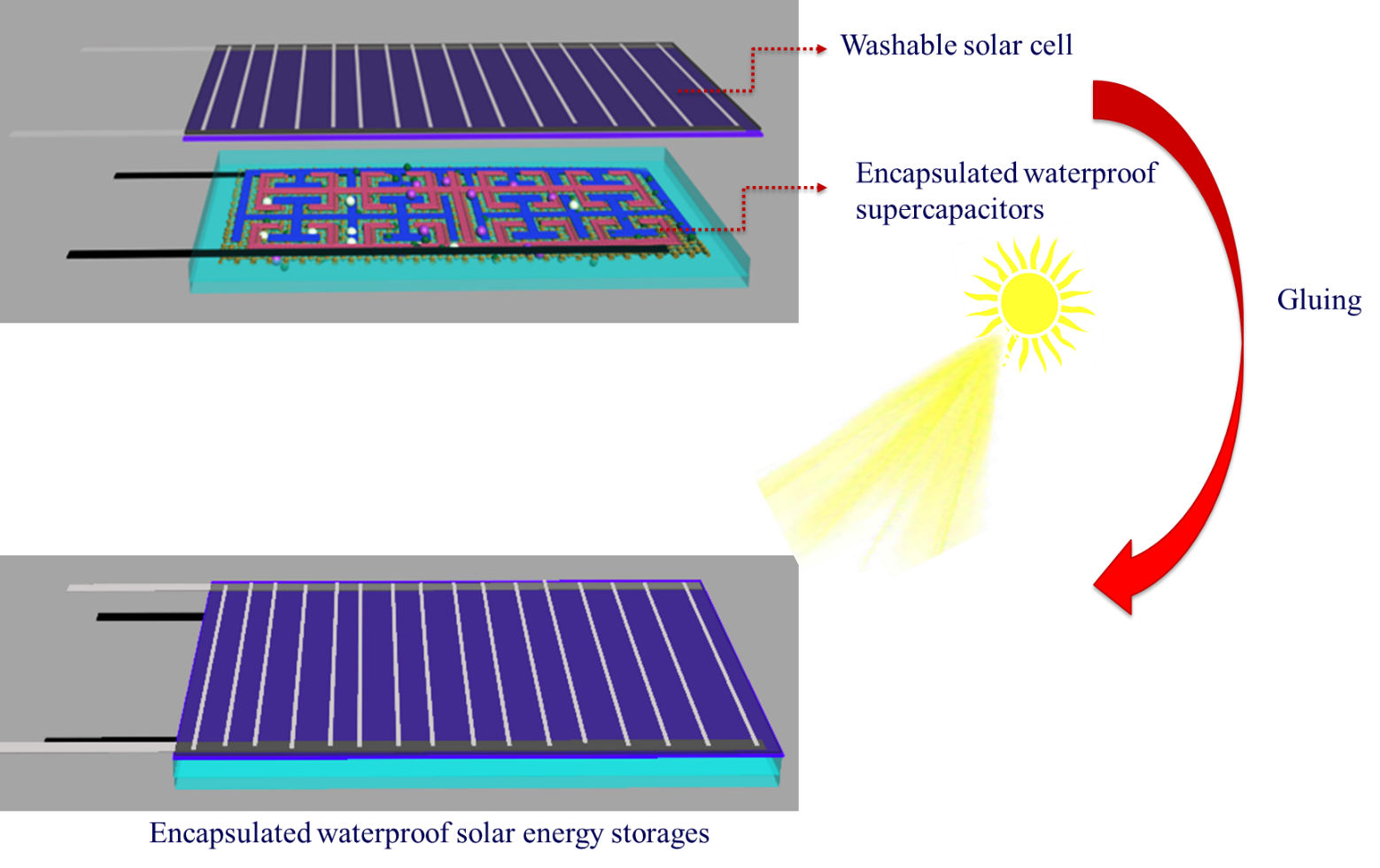


**Supplementary Figure 8**. Schematic of washable solar cell integration with the encapsulated waterproof laser-printed graphene supercapacitors.


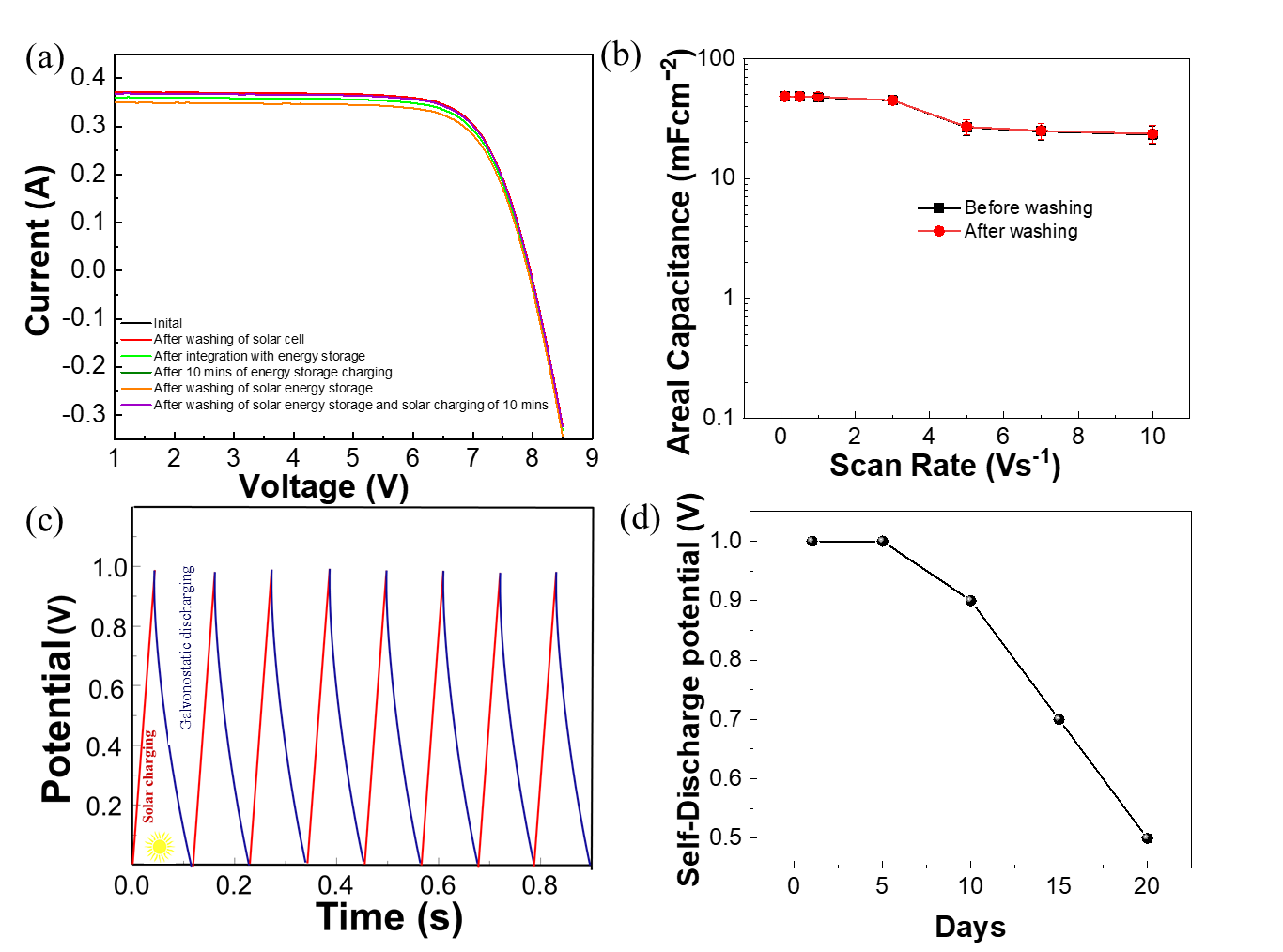


**Supplementary Figure 9.** **Characterizations of washable solar energy storage**. (a) Washable thin film amorphous silicon solar module performance before and after energy storage integration as well as the washing process at different periods. (b) Areal capacitance for the integrated energy storage with solar module before and after washing. (c) Solar charging-galvanostatic discharging cycles of the integrated energy storages. (d) Self-discharge curve for the textile integrated on-chip laser-printed graphene supercapacitors.

**References**

1 Thekkekara, L. V., Chen, X. & Gu, M. Two-photon-induced stretchable graphene supercapacitors. *Scientific Reports* **8**, 11722 (2018).

2 Conway, B. E. *Electrochemical supercapacitors: scientific fundamentals and technological applications*. (Springer Science & Business Media, 2013).

3 Lamberti, A., Clerici, F., Fontana, M. & Scaltrito, L. A Highly Stretchable Supercapacitor Using Laser-Induced Graphene Electrodes onto Elastomeric Substrate. *Advanced Energy Materials* **6**, 1600050 (2016).

4 Park, S., Lee, H., Kim, Y.-J. & Lee, P. S. Fully laser-patterned stretchable microsupercapacitors integrated with soft electronic circuit components. *NPG Asia Materials* **10**, 959-969 (2018).

5 Xu, R. *et al.* Kirigami-inspired, highly stretchable micro-supercapacitor patches fabricated by laser conversion and cutting. *Microsystems & Nanoengineering* **4**, 36 (2018).

6 Abdelkader, A. M. *et al.* Ultraflexible and robust graphene supercapacitors printed on textiles for wearable electronics applications. *2D Materials* **4**, 035016 (2017).

7 Bellani, S. *et al.* Scalable Production of Graphene Inks via Wet-Jet Milling Exfoliation for Screen-Printed Micro-Supercapacitors. *Advanced Functional Materials* **29**, 1807659 (2019).

8 Zhang, H., Qiao, Y. & Lu, Z. Fully Printed Ultraflexible Supercapacitor Supported by a Single-Textile Substrate. *ACS Applied Materials & Interfaces* **8**, 32317-32323 (2016).
